# How Tillage and Crop Rotation Change the Distribution Pattern of Fungi

**DOI:** 10.3389/fmicb.2021.634325

**Published:** 2021-06-17

**Authors:** Luigi Orrù, Loredana Canfora, Alessandra Trinchera, Melania Migliore, Bruno Pennelli, Andrea Marcucci, Roberta Farina, Flavia Pinzari

**Affiliations:** ^1^Council for Agricultural Research and Economics, Research Centre for Genomics and Bioinformatics, Fiorenzuola d’Arda, Italy; ^2^Council for Agricultural Research and Economics, Research Centre for Agriculture and Environment, Rome, Italy; ^3^National Research Council of Italy, Institute for Biological Systems, Rome, Italy

**Keywords:** agriculture, FUNGuild database, indicator value, mycorrhizae, soil, soil yeasts, rotation

## Abstract

Massive sequencing of fungal communities showed that climatic factors, followed by edaphic and spatial variables, are feasible predictors of fungal richness and community composition. This study, based on a long-term field experiment with tillage and no-tillage management since 1995 and with a crop rotation introduced in 2009, confirmed that tillage practices shape soil properties and impact soil fungal communities. Results highlighted higher biodiversity of saprotrophic fungi in soil sites with low disturbance and an inverse correlation between the biodiversity of ectomycorrhizal and saprotrophic fungi. We speculated how their mutual exclusion could be due to a substrate-mediated niche partitioning or by space segregation. Moreover, where the soil was ploughed, the species were evenly distributed. There was higher spatial variability in the absence of ploughing, with fungal taxa distributed according to a small-scale pattern, corresponding to micro-niches that probably remained undisturbed and heterogeneously distributed. Many differentially represented OTUs in all the conditions investigated were unidentified species or OTUs matching at high taxa level (i.e., phylum, class, order). Among the fungi with key roles in all the investigated conditions, there were several yeast species known to have pronounced endemism in soil and are also largely unidentified. In addition to yeasts, other fungal species emerged as either indicator of a kind of management or as strongly associated with a specific condition. Plant residues played a substantial role in defining the assortment of species.

## Introduction

Fungi dominate many habitats and have a central role as decomposers and key players in carbon, nitrogen and phosphorus cycles (Dighton, 2007). Mycorrhizal communities have a preeminent role in some ecosystems and contribute to defining plant community composition and fitness (van der Heijden et al., 2008). Thanks to the massive sequencing of fungal communities, some of the natural factors that most influence their structure and functionality have been unraveled in the last 20 years. Climatic factors, followed by edaphic and spatial variables, are among the best predictors of fungal richness and community composition at the global scale (Tedersoo et al., 2014). However, the analysis of anthropogenic activities and the effects of agricultural practices on fungal communities has been little investigated, and the main drivers in managed environments are less known. Fungal species inventories from agricultural soils are mainly known from classic culturing studies, while few studies employed cultivation-independent techniques so far (Klaubauf et al., 2010; Frąc et al., 2018). A deeper understanding of fungal communities’ composition, biodiversity and changes in agricultural soils is necessary to unveil what processes are carried out by members of these organisms. According to Stromberger (2005), the main contributions of the fungal community to the functioning of the agroecosystem are soil stabilization and nutrient cycling. Currently, many more roles have been attributed to fungi in the agroecosystems, like competitive or mutualistic interactions with bacteria or other frugivorous soil biotas and other networking functions that make fungal biodiversity a central element for the health of managed soils (Frąc et al., 2018). A primary pressure agricultural soil typically undergo is tillage, which can lead to homogenisation and structural degradation. Several studies have highlighted the effects of ploughing and the succession of events occurring in the transition from agricultural ecosystems subject to ploughing to more conservative systems (Six et al., 1999; Mikha and Rice, 2004; Seitz et al., 2019). These studies are essential for understanding the ecology of agroecosystems and, in particular, the role of fungi in the homeostasis of biogeochemical cycles and plant nutrition. These studies, however, based on biochemical indicators or culture-dependent methods were limited by the non-culturability of most fungal species (Mathew et al., 2012). Nevertheless, from such studies emerged that conversion from conventional tillage to no-tillage management implies a reduction in soil physical disturbance, and also a higher increase of fungal biomass, compared to bacterial biomass, as measured by the chemical indicator glucosamine (Joergensen, 2018), probably due to better maintenance of fungal hyphae integrity in soil (Miller and Lodge, 1997).

Moreover, several studies demonstrated an increase of arbuscular mycorrhizal (AM) fungi after the conversion of agricultural soil to no-tillage management, with the higher concentrations of glomalin positively correlated to soil macroaggregate stability (Wright et al., 1999; Curaqueo et al., 2010). Other variables affect the ecology and distribution of fungi in the passage between tillage to no-tillage, and vice versa. Soils under no-tillage tend to have cooler temperatures; the distribution of plant debris is different, air and water distribution are different, and so on. The changes in fungal communities caused by different soil management concerning ploughing are quantitative and qualitative. This is emerging especially more recently, thanks to molecular studies based on metagenomics and metabarcoding, but already in the past, it was evident that the mycorrhizal communities also changed in their composition. Tillage-resistant and tillage-sensitive AM species have been identified based on spores in soil, Non-Glomus AM fungal spores, including spores of *Gigaspora, Scutellospora*, and *Entrophospora* were detected more frequently in no-tilled soil, while *Glomus* spp. spores were retrieved in conventionally tilled soil (Jansa et al., 2002). According to Brito et al. (2012), in comparison with no-till, conventional tillage decreased AM fungal diversity by 40%, as detected by sequencing the DNA amplified from the large ribosomal subunit. Srour et al. (2020) studied with high-throughput sequencing the effects of long-term conventional tillage and no-till in parallel with three fertility treatments (No fertilization, N-only, and NPK) on soil microbial communities in a long-term field study that was established in the 1970s. They found that the tillage effect was more prominent than the fertilizer effect. Tillage significantly affected bacteria, fungi, fusaria, and oomycete beta-diversity, whereas fertilizer only affected bacteria and fungi beta-diversity.

In addition to ploughing, other types of management can have a macroscopic impact on the fungal communities of agricultural soils. For example, crop rotation exerts strong selective power on soil mycobiome structures (Sommermann et al., 2018) and is widely accepted to stabilize soil structure and fertility and control the spread of pathogens and weeds. As the shift to no-tillage reduces selective pressures on soil fungi, so does incorporate different crops into a rotation. Crop rotations increase plant diversity over time and, consequently, the heterogeneity of the available niches in the form of diverse substrate resources (Stromberger, 2005). The variables that determine the presence or absence from an ecosystem of a given fungal taxon can be truly infinite. Alternatively, they can be very few and decisive but challenging to circumscribe. Only a few studies showed that the occurrence of keystone fungal taxa could be explained by defined variables (soil phosphorus levels, bulk density, and mycorrhizal colonization) (Banerjee et al., 2019). In this study, the effects of tillage and crop rotation on fungal biodiversity and guilds distribution were analyzed in a long term experimental Mediterranean field, employing next-generation amplicon DNA sequencing, using a MiSeq sequencer and targeting as a fungal marker the internal transcribed spacer 1 (ITS1) region of the rRNA cistron. Fungal distribution in the different conditions was then read in the light of the chemical-physical characteristics of the soil. Moreover, the specificity of each OTUs to a given treatment was determined using the Dufrene-Legendre indicator value (IndVal) index (Dufréne and Legendre, 1997), while the FUNGuild database (Nguyen et al., 2016) was used to assign them to different ecological guilds.

## Materials and Methods

### Field Trial

The study site is located in the Foggia Province (Southern Italy, Apulia region, CREA experimental field, 41°27′57.3″N, 15°30′19.8″E, Thermo-Mediterranean climate), where mean annual temperature and rainfall of 15.8°C and 529 mm, respectively, are typically recorded (Di Bene et al., 2016; Castellini et al., 2019). Here, a long-term field experiment (1.2 ha) was established in 1995, aiming to monitor long-term effect of tillage and no-tillage management on durum wheat (*Triticum turgidum* subsp. *durum* Desf.) productivity. From 2009, a 2 year rotation with tick bean (*Vicia faba* L. var. minor) was introduced in the experimental design.

Since 2005, factors and levels of the trial were the following:

(1) Tillage

 (a) yes (T);

 (b) no (NT);

(2) Durum wheat/tick bean Rotation

 (a) yes (R);

 (b) no (NR).

T included a moldboard ploughing to 30 cm depth followed by a disc-harrow and flexible harrow seedbed preparation. In NT, no-tillage was carried out, and a soil seeding (with proper machinery) was conducted. The wheat straw was removed from the field after harvesting in both plots. The stubble was chopped and left on the surface of the NT plots white it was incorporated with tillage in the T plots. The thick bean plants were desiccated by a non-selective herbicide in May (full flowering), chopped and scattered on the soil surface in NT plot, and incorporated into the soil with the primary tillage in T plots (in autumn). Basal dressing in the T plots was carried out before the secondary tillage for seedbed preparation, providing 36 kg N ha^–1^ plus 96 kg P_2_O_5_ ha^–1^, as diammonium phosphate (18^–4^6^–0^). In the NT plot, the same fertilizer was spread on the surface. The seeding rate was set to 350 vital seeds m^–2^. Topdressing (64 kg N ha^–1^ as ammonium nitrate) was applied in both plots at the 21-29 tillering stage, according to BBCH scale (Hess et al., 1997).

### Soil Sampling and Chemical-Physical Analyses

In April 2018, at wheat and tick bean flowering, soil samples were collected in all 20 plots. Since the fungal communities in the rhizosphere soil are influenced mainly by the crop type, samples were collected from bulk soil using a standard cell sampling scheme (open polygons, 200 m^2^). Because of the long duration of the test, which made seasonal factors less relevant for statistical purposes, and the need to fully consider spatial variability, it was decided to sample only once but using a robust scheme with several replicates representing both chemical and biological variables (Nannipieri et al., 2019). 20 Plots (2 factors × 2 levels, *n* = 5) were then arranged accordingly in a factorial design on four strips of land with the following treatments: T-NR, NT-NR, NT-R and T-R. Samples were taken at 10 cm depth for both DNA analysis and chemical-physical characterisation. For DNA analyses, the samples were collected by inserting manually the falcon tubes (approximately 50 g) in the soil in order to reduce as much as possible soil manipulation; samples were hence immediately stored at –20°C. For the chemical-physical analyses, the samples (20 samples of approximately 1 kg each) were collected by using manual augers and introduced in labeled plastic bags for transportation and following processing (drying, sieving, and analyses) (Álvaro Fuentes et al., 2019).

All soil samples were analyzed for main chemical-physical parameters: soil texture by particle size analysis (like sand, silty, and clay percentage); bulk density (g cm^–3^); pH in KCl; total organic carbon (TOC g kg^–1^, LECO TOC Analyzer, mod. RC-612; LECO Corporation); total N (N_*tot*_ g kg^–1^, LECO Nitrogen analyser FP-528, St. Joseph, MI, United States); exchangeable K (K_ex_, in mg kg^–1^); bioavailable P, Fe, Cu, Zn, Pb, Cd, Ni, As (in mg kg^–1^), extracted by Mehlich 3 method (Mehlich, 1984; Ziadi et al., 1993) and analyzed by simultaneous plasma emission spectrophotometer (ICP-OES Iris; Thermo Optek, Milano, Italy). All tested soil parameters were analyzed by ANOVA, as affected by tillage (T), rotation (R), and T × R interaction. Means comparison was carried out according to *post hoc* Tukey’s HSD test using SPSS (IBM Corp., Armonk, NY, United States).

### DNA Extraction

DNA was extracted from 10 g of soil using the DNeasy PowerMax Soil Kit^®^ (Qiagen) following the manufacturer’s instructions. DNA crude extract yields were calculated using Qubi^®^t 2.0 Fluorometer (Invitrogen, Thermo Fisher Scientific, United States) following the manufacturer’s instructions. DNA quality was evaluated using Nanodrop^TM^ (Invitrogen, Thermo Fisher Scientific, United States). After extraction, the total amount of eluted DNA (5 mL) was purified by using Amicon Ultra 5 mL Centrifugal Filters 30K NMWL (EMD Millipore Corporation, Billerica, MA, United States). The purified and concentrated DNA was diluted to 10 ng μL-1 and used in downstream steps.

### Sequencing

Libraries were prepared using a modified version of the method proposed by Smith and Peay (2014). Sequencing libraries were generated by PCR amplification using locus-specific primers (ITS1f-ITS2) tailed with the Illumina adapters. The reverse primers were barcoded to allow multiplexing using the 12-base Golay barcodes (Caporaso et al., 2012). PCR amplification was performed in a final volume of 30 μl containing 3 μl of buffer 10X, 0.7 μl of each primer (10 mM), 0.9 μl of 50 mM MgSO4, 0.6 μl of 10 mM dNTP and 0.12 μl of Invitrogen Platinum Taq DNA polymerase High Fidelity (Cat N° 11304-011). The thermal cycling conditions used for the PCR were: initial denaturation at 95°C for 3 min followed by 35 cycles of denaturation at 95°C for 45 s, annealing at 50°C for 1 min and extension at 72°C for 1-min terminating with a final extension at 72°C for 10 min. Before sequencing, amplicon libraries were mixed with 10% PhiX control library to increase the sequences diversity. Libraries were sequenced on the Illumina MiSeq platform generating 300 bp paired-end reads.

### Bioinformatic Data Analyses

Raw reads were filtered using Trimmomatic (Bolger et al., 2014) to remove adapters and low-quality reads using a quality cutoff of 20 in 24 bp sliding windows. Paired-end reads were merged using Pear (Zhang et al., 2014). Reads were clustered into OTUs based on a 97% sequence similarity using the open reference OTU picking protocol implemented in the QIIME toolkit (Caporaso et al., 2010). Low abundant OTUs were removed, filtering out all the OTUs showing less than three sequences. Samples were normalized to the size of the smallest sample (67,511 sequences each) to allow comparison. Taxonomy was assigned using the UNITE database. The alpha diversity indexes, including Shannon (H) and Simpson (1-D) were calculated using the Vegan R package. The normal distribution of data within each condition was verified using the Shapiro Wilk test in R (V3.4.4). The ANOVA test and Tukey’s *post hoc* test was performed using the R package Stat 3.6.2. The Kruskal Wallis and the *post hoc* Dunn test with Benjamini Hochberg correction was performed using the R package dunn.test v 1.3.5.

Pairwise comparisons with the Wilcoxon rank test were run on R (V3.4.4). The distances between fungal communities were visualized using a NonMetric Multidimensional Scaling ordination derived from a Bray-Curtis dissimilarity matrix. The correlation between the soil texture properties and the NMDS ordination was tested using the *envfit* function in the R package Vegan. Statistical significance of community distance was tested using analysis of similarity (ANOSIM) implemented in the R package Vegan, with 999 permutations. The variables showing a significant correlation were plotted over the NMDS plot using the *ordisurf* function in the R package Vegan.

Different abundant OTUs in pairwise comparison between treatments were identified by using the edgeR package V 3.28.1 (Robinson et al., 2010). The threshold of statistical significance was defined by an adjusted *P*-value ≤ 0.05 and an absolute log2 fold change > 2. The specificity of an OTU to a given treatment was determined using the Dufrene-Legendre indicator value (IndVal) index (Dufréne and Legendre, 1997) using the labdsv package in R. “Indicator value” indices assess the predictive value of a species as an indicator of a combination of site groups. The indicator value index was calculated as the product of two quantities, A and B. “A” is the probability of a site being a member of a given site-group combination (T/NT soil combined with R/NR crops) when the species has been found at that site. Quantity “B” informs of how frequently the species is found at sites of the site-group combination under study. The “Correlation indices” assess the positive or negative preference of the species for the environmental conditions prevailing within sites belonging to the site-group combination, compared to the remaining sites.

FUNGuild^1^ database was used to assign ecological guilds to the OTUs (Nguyen et al., 2016). The FUNGuild software annotates taxonomic data within the OTU table with corresponding data on its database. The annotations include the guild trophic mode and growth morphology; confidence scores of “Probable” and “Highly Probable” were used. Prediction for functionality was based on assessments given in existing researches. The FUNGuild database divided the OTUs into 16 categories. However, for the alpha diversity analysis, we grouped within the broad category of saprotrophic fungi all the OTUs annotated, respectively, as leaf saprotroph, undefined saprotrophs, dung saprotroph and wood saprotrophs. Within the “saprotroph” class, we also considered the Mortierellaceae that were annotated by FUNGuild both as endophyte and saprotroph. The category “total mycorrhizal” was obtained grouping together the OTUs annotated as arbuscular mycorrhizal, ectomycorrhizal, and endomycorrhizal fungi by FUNGuild database. The Pearson correlation matrices were produced using the corrplot R package.

## Results

### Effect of Tillage and Rotation on Soil Chemical-Physical Properties

The effect exerted by T and R management and T × R interaction on investigated chemico-physical parameters is shown in Table 1. Bulk density increased in NT soils respect to T soils (*P* < 0.001), being also higher the sand percentage (+10%) compared to tilled soils (*P* < 0.05): without tillage, the soil texture shifted towards coarser soil particles, increasing the sandy component. No effect of rotation was observed on soil physical parameters.

**TABLE 1:** Effect of tillage (T/NT), crop rotation (NR/R) and factors’ interaction on main soil chemical-physical parameters.

	Bulk density	Sand	Silt	Clay	pH	TOC	N_tot_	P_av_
	g cm^–3^	%	%	%		g kg^–1^	g kg^–1^	mg kg^–1^
T-NR	0.93 b	29.5 b	50.7	19.7	7.85 ab	15.9	1.01 b	171.8 b
T-R	0.91 b	28.6 b	51.7	19.6	7.86 a	16.4	1.10 b	192.7 b
NT-NR	1.13 a	31.4 a	51.6	17.0	7.79 b	16.6	1.31 ab	240.7 ab
NT-R	1.22 a	31.8 a	52.1	15.9	7.79 b	16.6	1.28 a	311.2 a
Tillage (T) effect (Sig.)	***	*	n.s.	n.s.	**	n.s.	*	***
Rotation (R) effect (Sig.)	n.s.	n.s.	n.s.	n.s.	n.s.	n.s.	n.s.	*
T × R (Sig.)	n.s.	n.s.	n.s.	n.s.	*	n.s.	*	**

	**K_ex_**	**Fe_av_**	**Cu_av_**	**Zn_av_**	**Pb_av_**	**Ni_av_**	**Cd_av_**	**As_av_**

	mg kg^–1^	mg kg^–1^	mg kg^–1^	mg kg^–1^	mg kg^–1^	mg kg^–1^	mg kg^–1^	mg kg^–1^
T-NR	1559.7	42.8 b	4.3 b	8.6 b	18.1 a	0.92 b	0.29 a	0.55 ab
T-R	1512.5	65.1 b	5.2 b	6.5 b	12.4 ab	1.29 b	0.23 ab	0.50 b
NT-NR	1974.0	69.0 ab	5.4 ab	15.6 ab	7.3 b	1.61 b	0.19 b	0.55 ab
NT-R	1724.8	118.8 a	9.3 a	29.1 a	9.5 ab	3.92 a	0.21 ab	0.63 a
Tillage (T) effect (Sig.)	n.s.	n.s.	n.s.	*	n.s.	n.s.	n.s.	n.s.
Rotation (R) effect (Sig.)	n.s.	**	**	**	**	**	n.s.	n.s.
T × R (Sig.)	*	*	n.s.	*	*	n.s.	*	*

Levels of statistical significance are: *P < 0.05, **P < 0.01 and ***P < 0.001 (ANOVA). not statistically significant, n.s. Different letters represent significant differences (Tukey’s HSD test for means comparison). TOC = Total organic carbon, N_tot_ = total nitrogen, P_av_ = Phosphorus available, K_av_ = Potassium available, Fe_av_ = Iron available, Cu_av_ = Copper available, Zn_av_ = Zinc available, Cd_av_ = Cadmium available, Pb_av_ = Lead available, Ni_av_ = Nikel available, Cd_av_ = Cadmium available, As_av_ = Arsenic available.

The soil pH was significantly higher in T soils, ranging from 7.85 to 7.79 in NT ones (*P* < 0.01). pH was not significantly affected by rotation. The soil TOC was neither affected by both the considered factors nor by their interaction. The absence of tillage increased about 30% the N_*tot*_ compared to what recorded in tilled soils (*P* < 0.05); a little TxR interaction was observed, being the highest in NT-NR soils. Also, the available phosphorus (P_*av*_) was positively affected by the absence of soil tillage, being 35% higher in NT soils compared to T (*P* < 0.001), although a slight increase was observed under crop rotation (*P* < 0.05); such as observed for N_*tot*_, a moderate TxR interaction was observed on P_*av*_(*P* < 0.05), being the highest in NT-R soils. All nutrients and heavy metal contents (Fe_*av*_, Cu_*av*_, Pb_*av*_, Ni_*av*_, Cd_*av*_, and As_*av*_) were not influenced by tillage, with the exception of Zn_*av*_, which was triplicated in no-tilled soils with respect to the tilled ones (*P* < 0.05). On the opposite, micronutrients and heavy metals were affected only by crop rotation (*P* < 0.01), with the exception of Cd_*av*_ and As_*av*_, which were moderately affected by both the factors TxR (*P* < 0.05). The highest Fe_*av*_, Cu_*av*_, Zn_*av*_, Ni_*av*_ and As_*av*_ contents were recorded in NT-R soils, while the highest Pb_*av*_ and Cd_*av*_ were found in T-NR treatments.

### Effect of Field Management on Fungal Biodiversity

**FIGURE 1:**
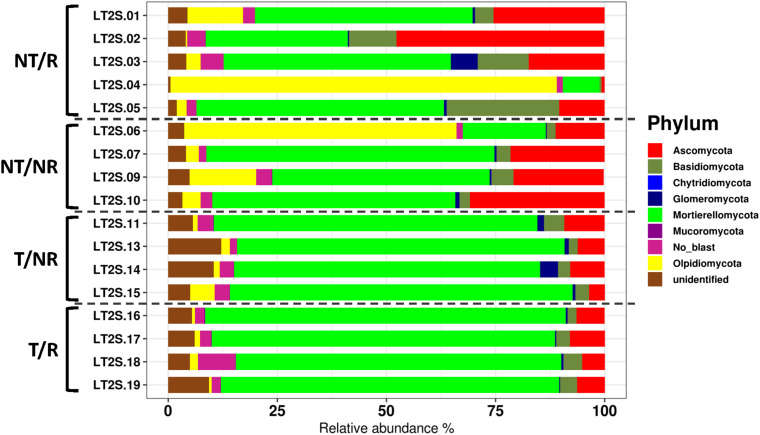
Barplot showing the fungal diversity at the phylum level.

**FIGURE 2:**
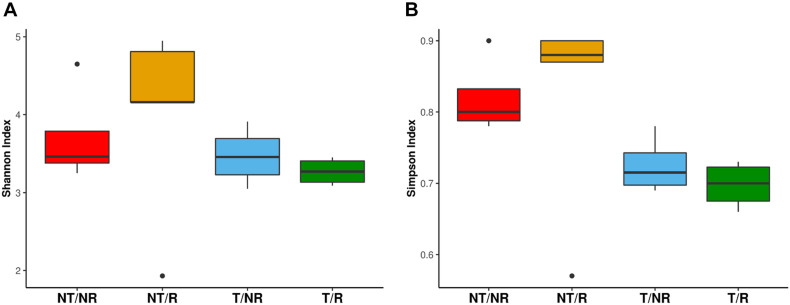
Boxplot showing **(A)** the fungal Shannon diversity index and **(B)** Simpson diversity index, measured in the four analyzed conditions: Conventional Tilling (T) and No tilling (NT) under continuous cropping (NR) system and Rotation (R).

The sequencing of the fungal ITS1 region from all soil samples yielded a total of 5223106 raw reads. After normalization, a total of 1646 OTUs were obtained (Supplementary Table 1). All the rarefaction curves (Supplementary Figure 1) calculated using the observed OTUs from each sample reached a plateau, indicating that the fungal biodiversity was fully captured in all the sequenced samples. The OTUs were assigned to 7 Phyla (Figure 1) and 284 different genera (Supplementary Table 2). The long-term field was dominated by Mortierellomycota (58% of sequences), followed by Ascomycota (14%), Olpidiomycota (12%) Basidiomycota (5.4%), Glomeromycota (1%), Mucoromycota (0.06%), Chytridiomycota (0.04%), and with 9.5% of OTUs assigned to unidentified fungal Phyla, and no blast hits (Figure 1). Alpha diversity measured using both Shannon and Simpson index did not show significant differences between treatments (Figure 2). However, as a general trend, samples from the fields under NT regime showed higher biodiversity, particularly in sites where crop rotation was applied (Table 2). The taxonomic composition of the soil fungal communities at the phylum level showed significant differences between fields managed with conventional T and NT. A significant higher presence of Ascomycota species was associated with the NT plot, while the Mortierellomycota and the Mucoromycota resulted significantly more abundant in conventional T field (Table 3). No significant differences were observed when samples were compared based on the rotation regimes.

**TABLE 2:** Summary of sequence data generated in this study and diversity estimate for each sample.

Sample	Condition	Raw	Trimmed	Observed	Shannon	Simpson
		reads	reads	OTUs		
LT2S.01	NT-R	354420	181261	593	4.16	0.88
LT2S.02	NT-R	262839	101736	713	4.81	0.90
LT2S.03	NT-R	207575	69024	631	4.95	0.90
LT2S.04	NT-R	489502	196390	417	1.93	0.57
LT2S.05	NT-R	341450	140384	511	4.16	0.87
LT2S.06	NT-NR	324655	175309	483	3.25	0.79
LT2S.07	NT-NR	353544	153390	576	3.50	0.81
LT2S.09	NT-NR	273369	110364	489	4.65	0.90
LT2S.10	NT-NR	267027	122597	596	3.42	0.78
LT2S.11	T-NR	257584	123462	586	3.62	0.70
LT2S.13	T-NR	250683	133027	491	3.29	0.73
LT2S.14	T-NR	322537	154775	563	3.91	0.78
LT2S.15	T-NR	278500	138195	509	3.05	0.69
LT2S.16	T-R	270039	138453	506	3.09	0.72
LT2S.17	T-R	315728	160514	520	3.45	0.73
LT2S.18	T-R	285386	140707	528	3.39	0.66
LT2S.19	T-R	368268	186022	498	3.15	0.68

*NT-NR = Durum wheat monocrop with no-tillage, NT-R = Rotation tick bean-durum wheat with no-tillage, T-NR = Durum wheat monocrop with conventional tillage, T-R = Rotation tick bean-durum wheat with conventional tillage.*

**TABLE 3:** Results of the Wilcoxon signed-rank test comparing the phylum abundance in Conventional tillage and no-tillage management systems.

Phylum	FDR
Ascomycota	**0.005**
Basidiomycota	0.37
Chytridiomycota	0.23
Entomophthoromycota	0.15
Glomeromycota	0.74
Mortierellomycota	**8.2 e^−05^**
Mucoromycota	**0.01**
Olpidiomycota	**0.02**
Rozellomycota	0.06
unidentified	**8.2 e^−05^**

*In the table are reported the FDR corrected P-value. Statistically significant values are shown in bold.*

### Fungal Community Responses to Soil Management

**FIGURE 3:**
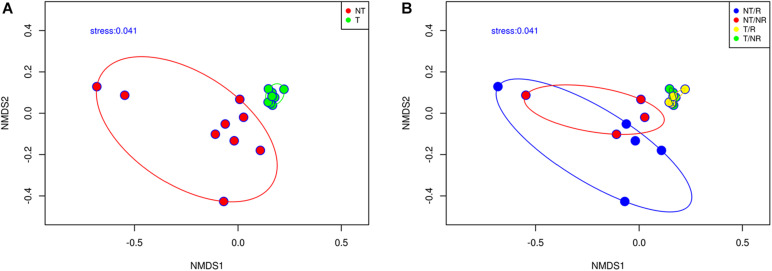
Non-metric multidimensional scaling based on Bray Curtis dissimilarities showing **(A)** the effect of tilling and **(B)** the effect of rotation on the fungal community composition.

We employed a Non-metric Multidimensional Scaling (NMDS) based on the Bray-Curtis dissimilarity matrix to analyze how the different soil management systems impact the fungal community structure (Figure 3). Samples from NT and T clustered in two significantly different groups (ANOSIM test *R* = 0.493; *P* = 0.001). Samples from T are clustered close to each other on the NMDS plot indicating high fungal community similarity, while the NT samples showed a higher dispersion on the NMDS bi-dimensional space (Figure 3A). The NMDS plot (Figure 3B) showed that the fungal communities from both R and NR cropping system clustered together in T systems. These results indicated that the soil fungal communities were shaped by T practices and only limitedly by crop R.

**FIGURE 4:**
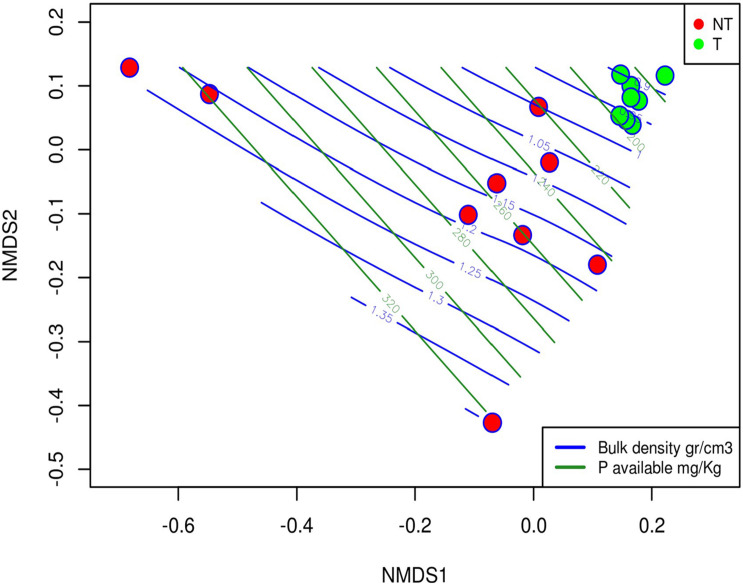
The NMDS plot overlaid with the bulk density and the phosphorus variables represented as isolines and showing the distribution of this variable over the samples.

The envfit function in the “Vegan R package” showed how the soil variables influenced fungal community composition. Ten variables that showed a significant correlation with the NMDS ordination (Table 4) were identified. Among them, bulk density (R^2^ = 0.84), the available zinc (Zn_*av*_, R^2^ > 0.72) and the available phosphorus (P_*av*_, R^2^ > 0.69) were the variables that resulted more correlated with the fungal community structure. To better display how the field management influenced soil properties and, in turn, the fungal community structure, the bulk density and the phosphorus soil variables were plotted as isolines over the NMDS ordination (Figure 4). These plots evidenced that fungal communities living in soil managed with T practices are immersed in an environment characterized by more homogeneous conditions from a physicochemical point of view. In contrast, the fungal communities from NT soils were distributed based on environmental conditions with high spatial variability (Figure 4).

**TABLE 4:** Envfit function output showing the variables significantly correlated with the NMDS ordination.

Variables	R^2^	*P*	
Bulk_Density	0.848	0.001	***
Sand	0.374	0.044	*
pH	0.388	0.037	*
TOC	0.298	0.879	n.s.
N_tot	0.913	0.442	n.s.
P_av_	0.693	0.002	**
Cu_av_	0.570	0.005	**
Zn_av_	0.724	0.002	**
Cd_av_	0.444	0.012	*
Pb_av_	0.556	0.006	**
Ni_av_	0.602	0.002	**
Fe_av_	0.553	0.006	**

TOC = Total organic carbon, Nt = total nitrogen, P_av_ = Phosphorus available, Cu_av_ = Copper available, Zn_av_ = Zinc available, Cd_av_ = Cadmium available, Pb_av_ = Lead available, Ni_av_ = Nikel available, Fe_av_ = Iron available. Levels of statistical significance are: *P < 0.05, **P < 0.01 and ***P < 0.001; n.s. means not statistically significant.

### Impact of Agricultural Practices on Fungal Functional Groups

**FIGURE 5:**
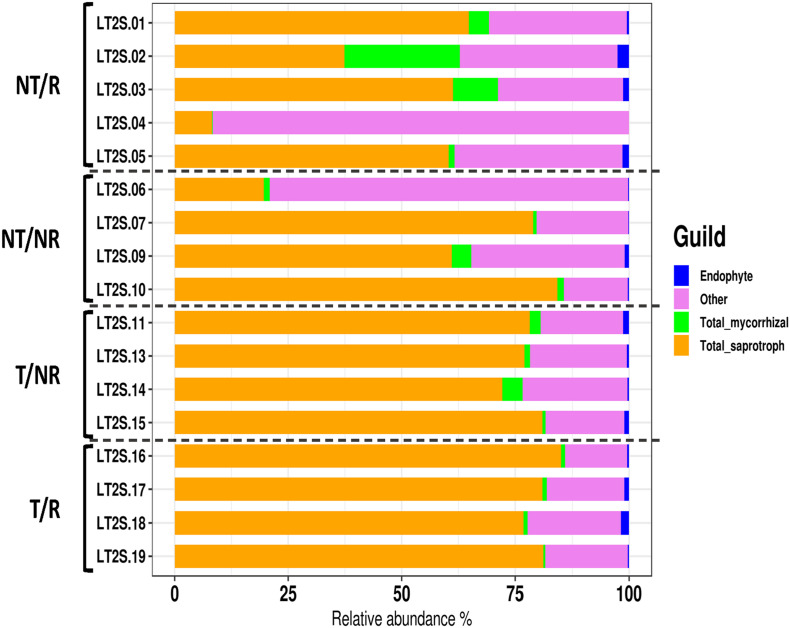
FUNGuild was applied to characterize the OTUs traits and analyze if the different soil management systems impact the main fungal functional groups such as mycorrhizal, saprotrophic and endophytic fungi. The barplot shows the relative abundance of the endophytic, mycorrhizal and saprotrophic fungi in the different samples.

**FIGURE 6:**
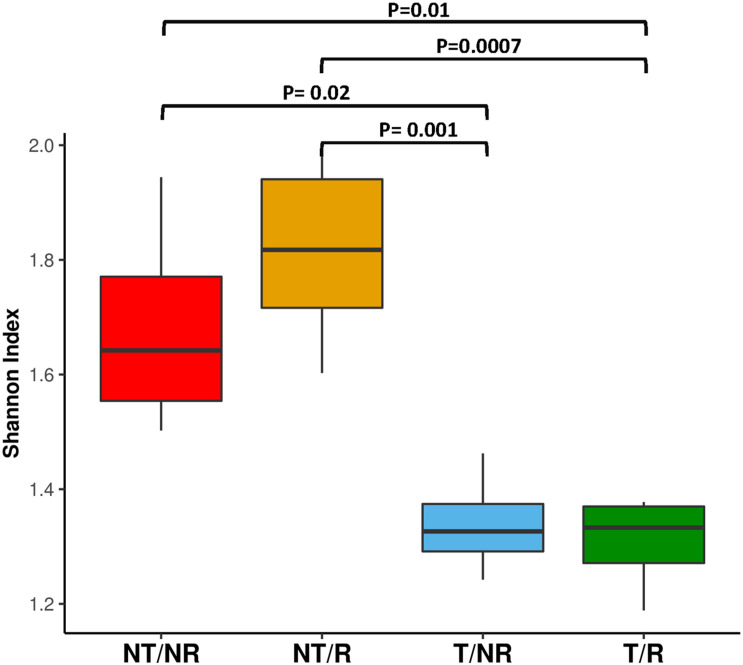
The boxplot shows the Shannon diversity index calculated on the OTUs classified as saprophytic fungi according to FUNGuild analysis.

FUNGuild was applied to characterize the OTUs traits and analyze if the different soil management systems impact the main fungal functional groups such as mycorrhizal, saprotrophic and endophytic fungi. As shown in Figure 5, saprophytic fungi dominated all the investigated soil conditions. The impact of T and NT agricultural practices on the biodiversity of saprotrophs, mycorrhizal and endophyte fungi was assessed using the Shannon index calculated within each functional group (Supplementary Table 3). The statistical analysis (Supplementary Table 4) revealed significant differences in the biodiversity of the saprotrophic fungi when rotation was applied. In this comparison, the soil under NT condition showed higher saprotrophic fungal biodiversity (Figure 6). No significant differences in biodiversity were observed between endophyte and mycorrhizal fungi.

Some soil properties influenced the biodiversity of saprotrophic and ectomycorrhizal fungi (Table 5). The biodiversity of saprotrophic fungi showed a positive correlation with soil bulk density (R^2^ = 0.82), bioavailable phosphorus (R^2^ = –0.67), sand percentage (R^2^ = 0.63) and some soil metals, such as Cd, Fe, Cu, Pb, Ni and Zn, measured as the bioavailable forms. A negative correlation emerged between the same fungal guilds and soil pH (R^2^ = –0.74). Differently from what shown with the saprophyte’s guild, the biodiversity of the ectomycorrhizal fungi showed a strong negative correlation with soil bulk density (R^2^ = –0.75), bioavailable phosphorus (R^2^ = –0.78) and a positive correlation with the soil pH (R^2^ = 0.68).

**TABLE 5:** Pearson correlation between the Shannon Index values of the ectomycorrhiza, the saprotrophic fungi and soil chemico-physical parameters.

	Total saprotroph		Bulk_Density		Sand		pH		P_av_		Cu_av_	
	R	P	R	P	R	P	R	P	R	P	R	P
Ectomycorrhizas	–0.8636	7.9E-06	–0.7539	0.0005	–0.6603	0.0039	0.6826	0.0025	–0.7892	0.0002	–0.7590	0.0004
Total saprotrophs	–	–	0.8229	0.0000	0.6380	0.0059	–0.7432	0.0006	0.6792	0.0027	0.6100	0.0093

	**Zn_av_**		**Cd_av_**		**Pb_av_**		**Ni_av_**		**Fe_av_**			
	**R**	**P**	**R**	**P**	**R**	**P**	**R**	**P**	**R**	**P**		

Ectomycorrhizas	–0.7421	0.0006	–0.6700	0.0033	–0.7647	0.0003	–0.8044	0.0001	–0.7667	0.0003		
Total saprotrophs	0.5979	0.0112	0.5015	0.0403	0.6132	0.0089	0.6810	0.0026	0.6210	0.0078		

*P_av_ = Phosphorus available, Cu_av_ = Copper available, Zn_av_ = Zinc available, Cd_av_ = Cadmium available, Pb_av_ = Lead available, Ni_av_ = Nikel available, Fe_av_ = Iron available.*

Furthermore, ectomycorrhizal fungi showed a significant negative correlation with some soil metals such as Cd, Fe, Cu, Pb, Ni, and Zn. The relationship between environmental variables and fungal guilds was then analyzed using the absolute number of reads counted within each ecological category (Table 6). In this way, we could evaluate whether the factor influencing the number of species also influenced the total abundance of each trophic guild. The Pearson correlation calculated on these data revealed an effect of metals concentrations on the abundance of saprotrophic fungi. In particular, the saprotrophic fungi abundance showed a negative correlation with bioavailable Cd, Fe, Pb, Ni, with a meaningful R value with Zn (R^2^ = –0.88) and Cu (R^2^ = –0.75). In addition, a negative correlation was also observed with the bioavailable phosphorus (R^2^ = –0.8), and the bulk density (R^2^ = –0.69).

**TABLE 6:** Pearson correlation between the abundance of the fungal guilds measured as reads number and the soil chemico-physical parameters.

	Bulk_Density		Sand		pH		TOC		Ntot		P_av_	
	R	P	R	P	R	P	R	P	R	P	R	P
Litter Saprotrophs	–0.7383	0.0007	–0.4216	n.s.	0.5148	0.0345	–0.5174	0.0334	–0.3674	n.s.	–0.8331	0.0000
Soil Saprotrophs	–0.6496	0.0048	–0.6809	0.0026	0.7724	0.0003	–0.3084	n.s.	–0.5103	0.0363	–0.7358	0.0008
Fungal Parasites	0.4497	0.0701	0.0130	n.s.	–0.1402	n.s.	0.3999	n.s.	0.1730	n.s.	0.6325	0.0064
Total Saprotrophs	–0.6991	0.0018	–0.2987	n.s.	0.3301	n.s.	–0.5308	0.0284	–0.1567	n.s.	–0.8073	0.0001
Total Mycorrhizas	0.5278	0.0294	0.6173	0.0083	–0.3946	n.s.	0.2594	n.s.	0.0880	n.s.	0.2844	n.s.

	**Cu_av_**		**Zn_av_**		**Cd_av_**		**Pb_av_**		**Ni_av_**		**Fe_av_**	
	**R**	**P**	**R**	**P**	**R**	**P**	**R**	**P**	**R**	**P**	**R**	**P**

Litter Saprotrophs	–0.7359	0.0008	–0.8850	2.3 e^−06^	–0.6873	0.0023	–0.7210	0.0011	–0.7232	0.0010	–0.6812	0.0026
Soil Saprotrophs	–0.6389	0.0058	–0.6161	0.0085	–0.5852	0.0136	–0.6198	0.0080	–0.6444	0.0052	–0.6732	0.0031
Fungal Parasites	0.5222	0.0315	0.7395	0.0007	0.5240	0.0309	0.5025	0.0398	0.4657	n.s.	0.4576	n.s.
Total Saprotrophs	–0.7542	0.0005	–0.8881	1.9 e^−06^	–0.7290	0.0009	–0.7410	0.0007	–0.7228	0.0010	–0.6796	0.0027
Total Mycorrhizas	0.3884	n.s.	0.2946	n.s.	0.3476	n.s.	0.4092	n.s.	0.4353	n.s.	0.3471	n.s.

*In the table are reported only the guilds that resulted statistically significant for p < 0.05. TOC = Total organic carbon, Ntot = total nitrogen, P_av_ = Phosphorus available, Cu_av_ = Copper available, Zn_av_ = Zinc available, Cd_av_ = Cadmium available, Pb_av_ = Lead available, Ni_av_ = Nikel available, Fe_av_ = Iron available. n.s. means that the correlation between the guild and the variable is not statistically significant.*

**FIGURE 7:**
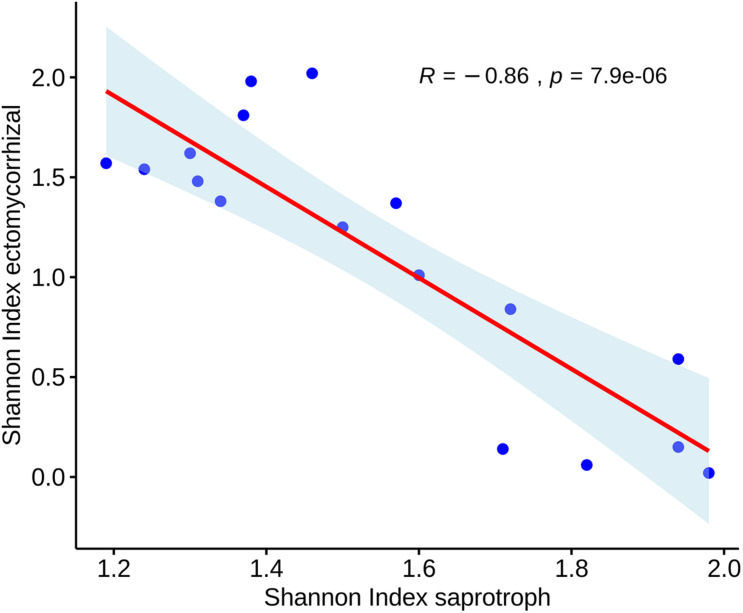
Scatterplot showing the Pearson’s correlation between biodiversity values (Shannon index) measured for ectomycorrhizal and saprotrophic fungi.

Total mycorrhizal abundance showed a positive correlation with bulk density (R^2^ = 0.5) and soil sand percentage (R^2^ = 0.61). Moreover, a significant inverse correlation (Pearson) emerged between the biodiversity (Shannon index) of ectomycorrhizal and saprotrophic fungi (Figure 7), but not between the corresponding abundances. Finally, the correlation between individual fungal taxa and the main soil parameters provided a few significant values. In particular, a negative correlation was observed between the abundance of fungal species of the genus *Mortieriella* with the bioavailable phosphorus (R^2^ = –0.83) (Supplementary Figure 2).

### Fungal Species as Indicators of Soil Management

**FIGURE 8:**
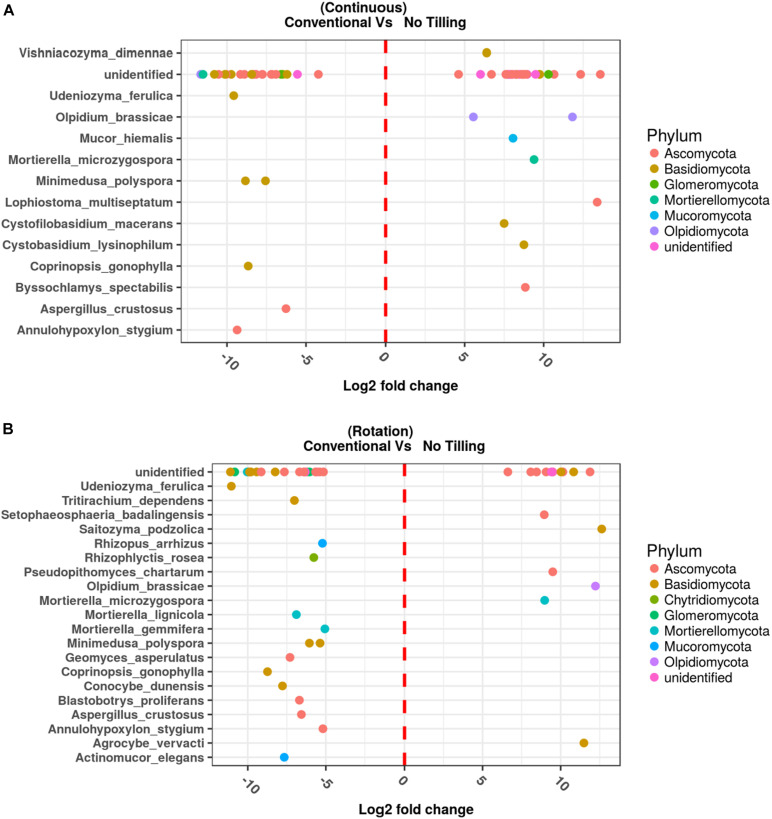
Differentially enriched operational taxonomic units assigned at the species level, assessed by EdgeR in the comparison between Conventional Tilling (T) and No Tilling (NT) under **(A)** continuous cropping system (NR) and **(B)** Rotation (R). Differentially enriched operational taxonomic units (OTUs) assigned at the genus, or higher taxonomic level are also reported in Supplementary Table 5.

**FIGURE 9:**
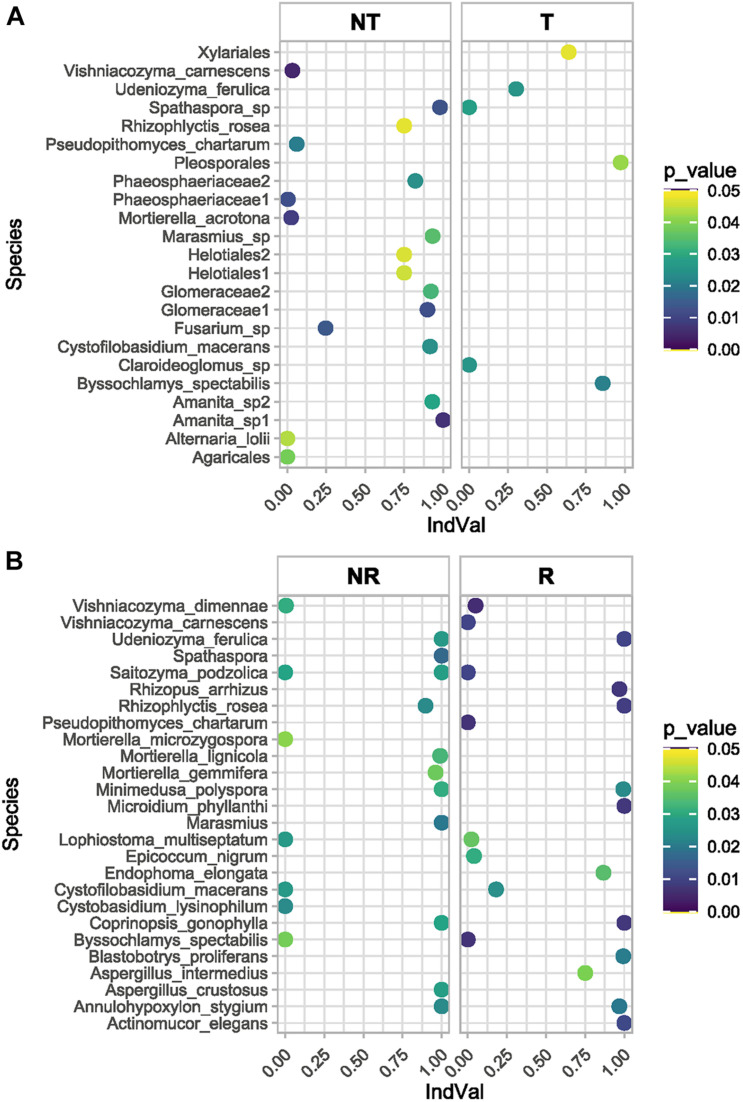
The indicator species analysis (see also Supplementary Table 6) assigned a significative indicator value (IndVal) to several unidentified OTUs. Among the identified OTUs the top indicator species of the site-group combination NR (T vs. NT) are shown in the plot **(A)**, while the top indicator species of the site-group combination R (T vs. NT) are shown in the plot **(B)**.

The fungal species associated with T and NT soils and R and NR crops were compared using two different approaches. The differential abundance of OTUs between pairs of conditions was calculated using edgeR (Robinson et al., 2010), and the results are reported in Figures 8A,B and the Supplementary Table 5. Moreover, to determine which OTUs could be used as indicators of a defined combination of agricultural soil managements (T-NT/R-NR), the indicator species analysis suggested by Dufréne and Legendre (1997) was applied, and the IndVal index between the species and each site-group calculated (Supplementary Table 6) to list the significant association values (Figures 9A,B).

The comparison between NT and T management under NR wheat cropping performed with Edge R (Figures 8A,B and Supplementary Table 5) showed some general results: (1) many differentially represented OTUs in all the conditions investigated were unidentified species, or OTUs matching at high taxa level (i.e., phylum, class, order); (2) both the degree of physical disturbance (T and NT) and the variability induced with rotation brought to significant differences in the soil fungal communities; (3) some fungal species were significantly associated to T or NT management no matter the use of crop rotation, while the opposite was not observed, no fungal OTUs were associated specifically to R or NR, independent of the T or NT condition.

Among the top fungal OTUs that were overrepresented in NT-NR management (combined condition with no-tilled soil and no-rotated crops), there was the OTU101, belonging to the Dothideomycetes with the highest LogFC value (13.58) and FDR = 0.0005, *Lophiostoma multiseptatum*, an OTU belonging to the Rutstroemiaceae, *Olpidium brassicae*, OTU14 belonging to the Glomeraceae with LogFC = 10.3 and an FDR = 0.0005, the basidiomycetous yeast *Saitozyma podzolica*, and with LogFC < 10 but significant FDR values, the species *Byssochlamis spectabilis, Cystobasidium lysinophilum, Cystophilobasidium macerans, Lophiostoma multiseptatum, Mortierella microzygospora, Mucor hiemalis, Olpidium brassicae, Vishniacozyma dimennae* along with other unidentified OTUs.

The top OTUs (Figures 8A,B and Supplementary Table 5) that were underrepresented in the NT-NR management, and conversely overrepresented in the T-NR combination (tilled, no-rotated) were the OTU107 corresponding to an unidentified Olpidiales with LogFC = –11.67 and FDR = 0.013, then a *Mortierella* species, an unidentified Cantharellales, two species of *Spathaspora*, an unidentified species belonging to the Ceratobasidiaceae family. Other identified species significantly overrepresented in the T-NR soil were: *Annulohypoxylon stygium, Aspergillus crustosus, Coprinopsis gonophylla, Minimedusa polyspora, Saitozyma podzolica*, *Udeniozyma ferulica.*

In the NT-R condition (no-tilled, rotated) (Figures 8A,B) the overrepresented OTUs (LogFC ≥ 10) corresponded to *Saitozyma podzolica* (logFC 12.63, FDR 0.0018), followed by *Olpidium brassicae*, then the OTU76 belonging to the phylum Ascomycota, unidentified and with a LogFC = 11.88 and an FDR = 0.002, the *Agrocybe vervacti*, an unidentified Agaricales, and the species *Lophiostoma multiseptatum*, *Mortierella microzygospora, Pseudopithomyces chartarum* and *Setophaeosphaeria badalingensis.* In the T-R soil (tilled, rotated) the OUT5, corresponding to an unidentified phylum, is the top ranked with LogFC = –12.27 and FDR = 0.019. This OTU was present in one sample with 2374 reads, and with 72 reads in another. Besides several unidentified basidiomycetes, there were several identified species significantly overrepresented in this condition: *Actinomucor elegans, Annulohypoxylon stygium Aspergillus crustosus, Blastobortys proliferans, Conocybe dunensis, Coprinopsis gonophylla, Geomyces asperulatus, Minimedusa polyspora, Mortierella gemmifera, Mortierella lignicola, Rhizophyctis rosea, Rhizopus arrhizus, Tritirachium dependens, Udeniozyma ferulica.*

Interestingly some species were overrepresented in the tilled soil (T) independent of crop rotation (R/NR) (Figures 8A,B). These were: *Annulohypoxylon Stygium, Aspergillus crustosus, Coprinopsis gonophylla*, *Minimedusa polyspora* and *Udeniozyma ferulica*, Instead, only one species was common to both the NT-R and NT-NR soils, the fungal species *Mortierella microzygospora*, which could therefore be considered representative of the no-tilled condition.

The indicator species analysis (Supplementary Table 6) assigned a significative indicator value to several unidentified OTUs. Among the identified OTUs (Figures 9A,B) the top indicator species of the site-group combination NR (T vs. NT) were: *Annulohypoxylon stygium, Cystobasidium lysinophilum, Cystofilobasidium macerans*, a species of *Spathaspora*, *Lophiostoma multiseptatum*, a species of *Marasmius*, *Minimedusa polyspora, Rhizophlyctis rosea, Udeniozyma ferulica.* Among the top indicator species of the site-group combination R (T vs. NT) there was again a *Marasmius* species, and the fungal species: *Byssochlamys spectabilis, Coprinopsis gonophylla*, *Microidium phyllanthi, Pseudopithomyces chartarum, Rhizopus arrhizus, Vishniacozyma dimennae.* In the site-group combination NT (R vs. NR) the top indicator species were an *Amanita* species, *Cystofilobasidium macerans, Mortierella acrotona*, *Pseudopithomyces chartarum* and *Vishniacozyma carnescens*. The top indicator species in the site-group combination T(R vs. NR) were *Byssochlamys spectabilis*, a *Claroideoglomus* species, a *Spathaspora* species and *Udeniozyma ferulica.*

## Discussion

### Impact of Soil Management Systems on Biodiversity and Communities’ Distribution

We have evaluated the impact on fungal communities of two contrasting ploughing management systems both with and without crop rotation. The analyses performed measuring both the Shannon and the Simpson diversity indexes did not show significant differences at the ANOVA test. However, as shown in Figure 2, the biodiversity tended to be higher in fields under conservative management, being increased in NT (no-tilled) soils, where R (rotation) was applied. The differences were greater using the Simpson index, known to be more weighted on common or dominant species. The more pronounced differences observed with the Simpson index than Shannon biodiversity in NT soil may indicate that soil T disturbance is less affecting species richness but has a higher impact on common or dominant species. The latter, instead, appeared more preserved by the adoption of conservative management.

The inverse correlation that emerged between the biodiversity (Shannon index) of ectomycorrhizal and saprotrophic fungi (Figure 7) could have an ecological significance and be linked to the niches differently available in the contrasting soil management systems.

Bödeker et al. (2016) suggested that saprotrophic and root-associated fungal guilds have overlapping fundamental niches for colonizing substrates of different qualities. The authors found that in boreal forest soil profiles, mycorrhizal fungi, through competitive interactions, can indirectly regulate litter decomposition rates by restraining activities of more efficient litter saprotrophs. According to the authors, substrate-dependent depth partitioning in forest soils of ectomycorrhiza-dominated ecosystems is reinforced by interference competition. Agricultural soils have very different niche partitioning dynamics; however, Figure 7 indicates that saprotrophic and root-associated fungal guilds exclude each other in agricultural cropping soil. The inverse relationship between ectomycorrhizal and saprotrophic fungi was, in our case, based on biodiversity values, while the mere abundance of the two ecological groups seemed unrelated. The partitioning between ectomycorrhizal and saprotrophic fungi could be due to substrate-mediated niche partitioning (i.e., distinct fundamental niches), or space partitioning (i.e., distinct realized niches, as in-depth partitioning). Spatial partitioning of these fungal guilds could be particularly critical in agricultural soils, as they regulate carbon and nutrient cycling in different ways (Bödeker et al., 2016; Soudzilovskaia et al., 2019). The absence of soil disturbance and high raw carbon sources availability, as in NT soil, could have favored the presence of saprophytic fungi in topsoil (Figure 6). Although Sun et al. (2020) verified how tillage treatment changed the vertical distribution of fungal communities in soil, particularly in terms of relative abundance of the species at the different depths, a different accumulation of crop residues in T and NT soils could better justify the spatial partitioning of species. The decomposition of high C/N residues is generally dominated by saprophytes, whereas roots-associated mycorrhizal fungi colonize deeper soil (Bödeker et al., 2016). The long term no-tillage management could have modified the mineralisation kinetics of organic crop residues, typically rich in lignin (about 15% in wheat straw) and cellulose (up to 70-75%) favoring saprophytic lifestyle at the detriment of ectomycorrhizal species, which prefer N-rich ready-to-use substrates (Hobbie and Horton, 2007; Sharma-Poudyal et al., 2017). In tilled soils, crops’ residues have been constantly buried and moved into deeper layers, limiting their colonization and decomposition by saprotrophic species.

The effect of the two agricultural soil management systems was analyzed considering the influence of the different tillage practices on the soil’s physicochemical properties and, in turn, their consequent impact on fungal community structure. Bulk density is an indicator of the total porosity of the soil: the lower it is, the higher is the porosity, and the higher is the water that can infiltrate and be stored in the soil. In our systems, the absence of tillage modified the soil texture in the long term, increasing the sandy fraction and slightly decreasing the pH. As reported by Kabir et al. (1997), fungal hyphal density and fungal symbiosis generally increase in well-aggregated soil, with good gas exchange, water infiltration, high water, and nutrient storage capacity, thus offering heterogenous microsites and favoring soil fungal and microbial diversity (Blevins et al., 1984). Although no correlation emerged among soil chemical-physical parameters and ectomycorrhizal biodiversity (Table 5), a positive correlation between the abundance of total mycorrhizal fungi and the soil bulk density, such as with the soil sandy fraction, was found (Table 6).

We speculated that different mycorrhizal species are differently sensitive to soil compaction and reduced pore space: it was found that the genus *Funneliformis*, the most abundant AMF across all land-use types, characterized by short life cycles, were less sensitive to the disruption of the extraradical mycelia that is sustained in tilled soils. In contrast, the species of the genus *Scutellospora*, primarily dependent on host plants presence, resulted sensitive to extraradical mycelia disruption caused by frequent tillage (Pellegrino et al., 2020). Furthermore, Gigasporaceae typically prefer bulk soil rich in pores suitable to the fungal hyphal development (Maherali and Klironomos, 2012), while the *Glomus* and *Rhizophagus* genera mainly develop their hyphal biomass in the roots, more than in the bulk soil.

In our systems, the increase of total mycorrhizal fungi abundance in no-tilled soils results from species selection by the higher soil compaction and lower pore aeration, which increases physical resistance to hyphal development and penetration. Some fungal species, such as Gigasporaceae (i.e., Glomeromycota), have been probably favored in no-tilled soils where the sandy fraction was higher, thus justifying the recorded increase of total mycorrhizal fungi abundance.

Crop rotation played a crucial role in influencing the soil nutrient stocks: introducing the tick bean into the crop rotation led to an increase in the soil nitrogen supply due to legume N-fixing activity. In addition, iron, copper, and zinc, essential co-factors in soil enzymatic activities (Cabrera et al., 2003; Mat Hassan et al., 2012), also remained in the soil after tick bean cropping. However, in the studied systems, the total abundance of mycorrhizal fungi was correlated mainly with the soil’s physical structure (Table 6).

The absence of soil disturbance and the high nutrient availability enhanced saprophytic fungi biodiversity (Figure 6). Different accumulation of crop residues in T and NT soils may justify this result: decomposition of high C/N residues is generally dominated by substrate degrading saprophytes, whereas mycorrhizal, beneficial fungi colonize mostly soils containing humified organic matter (Bödeker et al., 2016). The no-tillage regime could have lowered the mineralisation of organic crop residues in the upper organic soil layer to favor saprophytes growth more than fungi unable to decompose lignin and cellulose-rich substrates (Hobbie and Horton, 2007).

Another noteworthy aspect is the different degree of data variability in the two systems, NT and T (Figure 3A). From Figure 3A, we can conclude that where the soil was tilled, the species were evenly distributed, while in the absence of tillage, there was high spatial variability. The fungi were linked to small scale micro-niches, heterogeneously distributed, that remained undisturbed due to the absence of ploughing. The NMDS plot in Figure 3B showed that the communities from both R and NR systems clustered together within each tillage practice. The lack of a clear differentiation between the soil fungal communities’ profiles associated with rotation and continuous cropping systems could be partially explained considering that the analysis was made in the bulk soil. Plants select their microbiome by releasing roots exudates, and microorganisms form complex communities in the rhizosphere that are strongly influenced by the plant roots (Schloter et al., 2018). Bulk soil may represent a sink of microbial diversity from which roots recruits the rhizosphere microbiome. The effect of plant roots could be temporary and limited to the rhizosphere. At the bulk soil level, roots could play a minor role in shaping fungal communities, which could explain the lack of detectable effects of rotation on the fungal community’s beta diversity. The overall results indicate that different soil management systems influence the soil texture and the chemical attributes creating contrasting microhabitats that support different fungal communities.

### Differential Responses of Soil Fungi to Management Systems

Among the fungal species that seem to have played a role in defining the communities developed under different soil management, there are several yeasts. Soil yeasts display pronounced endemism along with a high proportion of currently unidentified species (Yurkov, 2018).

Many yeast species live as saprophytes in soil utilizing a broad spectrum of carbon sources ranging from simple sugars to complex polymers like cellulose, hemicellulose and phenolic compounds produced by the hydrolysis of lignin. Yeasts can influence soil aggregation by producing extracellular polysaccharides, and some species were found to be able to promote plant growth, producing bioactive compounds in the rhizosphere (Mašínová et al., 2017).

A significant yeast species individuated in this study is *Saitozyma podzolica*, with OTUs significantly overrepresented in all the conditions except the tilled soil with crop rotation (T-R). This species showed the highest logFC value in the NT/R samples, which makes this organism associated with the most conservative soil management (Figure 8B). *Saitozyma podzolica* (synonym of *Cryptococcus podzolicus*) is an oleaginous yeast that can convert xylose to oil. It has been reported as a candidate for maintaining the carbon value chain by converting renewable waste material, e.g., hydrolyzed wood and straw, for biodiesel production (Schulze et al., 2014). Interestingly Buee et al. (2009) studied some forested soils in France and showed that *Saitozyma podzolica* was among the species with the highest sequence abundance. Also, Yarwood et al. (2010) identified *Saitozyma podzolica* as one of the most abundant taxa in forest soil in Oregon, United States. This yeast is in the Basidiomycota clade and belongs to the Tremellaceae family, in the order Tremellales. *Saitozyma podzolica* showed biological control ability against post-harvest pathogens in apples, such as *Penicillium expansum* (Wang et al., 2018). This indicated that the fungus could have the ability to prevail in soil over other fungal species. Another yeast that was overrepresented in different conditions is *Udeniozyma ferulica*. The genus “*Udeniozyma”* has been proposed in Wang et al. (2015) to accommodate species previously attributed to other genera, among which the genus *Rhodotorula* in which the species was classified as *Rhodotorula ferulica*. This yeast can use as a single carbon source many phenolic compounds: vanillyl alcohol, vanillic acid, veratryl alcohol, veratric acid, ferulic acid, p-hydroxybenzoic acid, m-hydroxybenzoic acid, protocatechuic acid, p-coumaric acid, and caffeic acid (Sampaio and Van Uden, 1991), which makes it a species remarkably capable of assimilating intermediates of lignin degradation (i.e., ferulic, 4-hydroxybenzoic and vanillic acids) (Yurkov, 2018). *U. ferulica* was overrepresented in tilled soils, with and without crop rotation. Moreover, it was among the indicator species in two site-group combinations: NR (T vs. NT) and T (R vs. NR). It seems that this fungus is favored by disturbance and stressing conditions, such as that of a monoculture of wheat, which is also a condition with a constant input of straw, that is buried with tilling and made part of the lignin-rich substrate available for microbes in the soil.

An unidentified species of *Spathaspora* showed a similar distribution as *U. ferulica.* In fact, it was overrepresented in tilled soils, with and without crop rotation, and it was among the indicator species in two site-group combinations: NR (T vs. NT) and T (R vs. NR), just like the other yeast. *Spathaspora* genus comprehends yeasts that can convert d-xylose to ethanol (Cadete and Rosa, 2018). Interestingly, most of the species described so far in this genus have been isolated from rotten wood or in the gut of beetles’ larvae eating wood (Cadete and Rosa, 2018). According to Cadete and Rosa (2018), the midgut of wood-boring beetles is hypothesized to be oxygen-limited, so adaptation of species of *Spathaspora* for growth under oxygen-limited conditions on mixtures of cellulosic and hemicellulosic sugars might be expected (Long et al., 2012). The fact that *Spathaspora* species are prone to live on cellulosic remains, utilizing xylose in conditions of microaerophilia, reinforces the hypothesis that these yeasts can benefit from the burying of wheat straw through tilling practices. Two species of *Vishniacozyma* genus showed an indicator species role (Figure 9): *Vishniacozyma carnescens*, indicator species of site group combination NT (R vs. NR) and *Vishniacozyma dimennae* indicator species of site group combination R (T vs. NT) (Liu et al., 2015). The genus *Vishniacozyma* produces starch-like compounds and can utilize galactose, trehalose, and cellobiose, but cannot assimilate nitrate, methanol, or ethanol (Boekhout et al., 2011). The type strain of *Vishniacozyma dimennae* had been isolated from pasture plants in New Zealand. It is a species lacking the ability to utilize sugars with α glucosidic bonds, except trehalose; it has been described based on its inability to utilize nitrate, maltose, and melezitose (Fell and Phaff, 1967). Overrepresented in NT-NR soil and indicator species of site group combination NR (T vs. NT) were *Cystobasidium lysinophilum* and *Cystofilobasidium macerans*. *Cystofilobasidium macerans* was indicator species also of the site group combination NT (R vs NR). Both are yeasts of the class Basidiomycetes, which in general have agricultural and medical importance because several species have an impact on bio-control of plant diseases, and others can break down aromatic compounds and are considered useful in bioremediation, or are capable of accumulating lipids in lipid bodies that can account for up to 65% of their dry biomass.

Moreover, both these species are cold-adapted. A strain of *Cystofilobasidium macerans* showed both cellulolytic and proteolytic activity (Chreptowicz et al., 2019). The ability to utilize various carbon sources, if combined with the production of extracellular hydrolytic enzymes, is particularly functional to the processing of agricultural wastes such as lignocellulosic biomass in the form of straw, which contains polymers composed of sugars like glucose, xylose, galactose, or cellobiose (Chreptowicz et al., 2019). The ecology of *Cystobasidium lysinophilum* is very particular. *Cystobasidium* was described as a genus of mycoparasites of coprophilous ascomycetes (Sampaio and Oberwinkler, 2011). Finally, the yeast *Blastobotrys proliferans*, in the Saccharomycetales order, was significantly overrepresented in the tilled soil with crop rotation. This osmotolerant ascomycetous yeast is resistant to high metals and salts concentrations (Mukherjee et al., 2017).

In addition to yeasts, several other fungal taxa have shown an interesting distribution related to the type of soil management. Among the most representative and interesting species that have shown an important role in defining the differences between the soil communities of contrasting soil managements, *Minimedusa polyspora* (Pinzari et al., 2014) showed an overrepresentation in the tilled soil, independent of the rotation of crops. The fungus was characteristic of T-NR and T-R soils and was one of the top indicator species of the site group combination NR(T-NT). *M. polyspora* is a filamentous basidiomycete that has previously been isolated mainly from the soil, and straw, wood, old paper, oat seed, and cotton flowers with a worldwide distribution (Větrovský et al., 2020). Culture-independent surveys of fungal diversity in arable and grassland soils revealed in the last 10 years a high abundance of *M. polyspora* (Klaubauf et al., 2010; Větrovský et al., 2020). This fungus is cellulolytic, and it prefers polysaccharides at the initial phases of its growth and hexoses or oligosaccharides in the later phases of its development. It is a very efficient pioneer colonizer that requires little nitrogen, is fast-growing, changes its metabolism according to the first modifications of the substrate, and uses inhibitory substances to make the habitat unfavorable for other species (Pinzari et al., 2014), which makes it fit for disturbed and stressed agrarian soils.

Another species showing the same pattern of presence and role of *M. polyspora* is *Annulohypoxylon stygium* (Xylariales, Ascomycota), a white-rot fungus commonly found on dead wood (Liu et al., 2019). This fungus, like *M. polyspora*, was overrepresented in T-NR and T-R soils and was an indicator species of the site group combination NR(T-NT). *A. stygium* shows exceptionally high performance in lignin and carbohydrate degradation (Liu et al., 2019). Interestingly, this fungus was identified to be the main symbiotic species of *Tremella fuciformis*, medicinal and culinary fungi of China which is a fungus that does not form an edible basidiome without the presence of a specific host fungus both in nature and for industrial production (Liu et al., 2019). *A. stygium* co-occurred in the tilled soil with some unidentified species of the Tremellales order. There were other fungal species significantly overrepresented in tilled soil, with both rotated and not rotated crops. These were *Coprinopsis gonophylla*, which is widespread but, according to Redhead et al. (2001), rare in Europe, and it grows solitary or fasciculate on burnt places or bare, clayey soil. *Aspergillus crustosus*, a fungus known to produce PR-toxin (7-acetoxy-5,6-epoxy-3,5,6,7,8,8a-hexahydro-carboxaldehyde), a bicyclic sesquiterpene that belongs to eremophilane (a terpenoid) on cereals and forages (Varga et al., 2010). Interestingly, *A. crustosus* is the only *Aspergillus* identified at the species level and significantly overrepresented in tilled soil. In contrast, the expected frequency of *Aspergillus* species in agricultural soil is definitely higher, based on counts of *Aspergillus* species reported in over 250 studies of microfungi from soils and litter. However, little or no distinct pattern of *Aspergillus* species occurrence was found for the forest, wetland, or cultivated soils (Klich, 2002). A group Mucoromycotina fungi, members of the genera *Rhizopus*, *Actinomucor*, and *Mortierella* were significantly overrepresented exclusively in the tilled soil with crop rotation. *Mortierella* species are fungi belonging to the order Mortierellales within the subphylum Mortierellomycotina (phylum: Mucoromycota). Most species are saprobic soil-inhabiting with the ability of diverse biotransformations or the accumulation of unsaturated fatty acids. *Mortierella* species have been found living as endophytes in plant’s roots (Urbina et al., 2018), and others showed the ability to solubilise phosphatic rocks, making P available to plants (Ceci et al., 2018). Two species associated with tilled soil were identified as *Mortierella lignicola* and *Mortierella gemmifera*, while *Mortierella microzygospora* was significantly associated with the no-tilled soil. Members of the genus *Mortierella* were abundantly represented in other Next Generation Sequencing (NGS) investigations of soil fungi, featuring amongst the top ten taxa in arable soils (Detheridge et al., 2016). It is noteworthy that the genus *Mortierella*, which comprehend several phosphate-solubilising species (Zhang et al., 2011; Ceci et al., 2018) was negatively correlated with the concentration of available phosphorus in the soil (Supplementary Figure 2). This seems to indicate a distribution of the species of this genus in a particular trophic niche, characterized by the low availability of phosphorus, probably limiting the presence of other competitor species.

Other Mucoromycotina overrepresented in the tilled soil were *Rhizopus arrhizus* and *Actinomucor elegans*. *Actinomucor* species are found in dung, soil, food, and human sources. *A. elegans* is considered a source of glycine aminopeptidase and glucosamine (Wang et al., 2014).

*Mucor hiemalis*, a Mucoromycotina fungus that can grow below 0°C, was instead overrepresented in no-tilled soil, where bioavailable Cu, Zn, Ni, and As were higher than in the tilled one. This species is a biotechnologically relevant fungus, recently studied for its extraordinary metal/metalloid bioaccumulation capacity (Hoque and Fritscher, 2019).

*Olpidium brassicae* was strongly associated with no-tillage soil management independently of the crop rotation, in fact it showed higher logFC in both NT-NR and NT-R soils. *O. brassicae* is a soil-borne fungus within the phylum Chytridiomycota, a root-infecting obligate plant parasite, widespread in temperate regions (Hilton et al., 2013). Other authors have also noted the effect of no-tillage on the persistence and abundance of certain pathogenic fungi. For example, Wang et al. (2020) observed that relative abundances of some plant pathogenic fungal genera increased significantly under no-tillage treatments. Another species representative of no-tillage management was *Byssochlamys spectabilis*, whose anamorph is *Paecilomyces variotii*. This cosmopolitan fast-growing thermotolerant fungus can grow at low oxygen levels and in the presence of preservatives, form the mycotoxin viriditoxin, and be able to survive heat treatment. It is found in soils, indoor environments, plants, animals, and foodstuffs (Houbraken et al., 2008).

Interestingly *B. spectabilis* was also an indicator species of the site group combinations R(T-NT) and T(R vs. NR). *Lophiostoma multiseptatum* is also overrepresented in no-tilled soil, independent of crop rotation (NT-NR + NT-R). Moreover, it is an indicator species of the site group combination NR(T-NT). *Lophiostoma* is a genus of ascomycetous fungi in the family Lophiostomataceae that are species commonly found growing both on living and dead wood, the bark of deciduous trees, on shrubs, and herbaceous hosts (Mugambi and Huhndorf, 2009).

An interesting species overrepresented in no-tilled rotated soil is *Pseudopithomyces chartarum*, known to cause leaf spot diseases of various plants, including medicinal plants, grasses, cereals, legumes. It has been recorded as a wheat pathogen. In addition, some isolates of *P. chartarum* produce sporidesmin, a hepatoxic mycotoxin that causes skin damage to sheep, goats, and cattle (Perelló et al., 2017).

Finally, although not overrepresented in any specific condition, some fungal species were indicator species of a site group combination. This is the case of unidentified species of the basidiomycetes genera *Marasmius* and *Amanita*, indicator species of site group combination R (T vs. NT) and NT (R vs. NR), respectively. Moreover, the fungus *Rhizophlyctis rosea* (Rhizophlyctidales, Chytridiomycota) was an indicator species for the site group combination NR (T vs. NT). *R. rosea* is one of the most frequently observed zoosporic fungi in cropping soils where, according to Marano et al. (2011), it may play an essential role in the degradation of cellulosic debris. *R. rosea* appeared to be slightly more common in disturbed (e.g., cropping systems) than in natural soils, indicating adaptation to stressors, such as a wide range of temperatures, associated with soil cultivation (Marano et al., 2011).

A few OTUs assigned to the phylum Glomeromycota, identified at the family level and in one case at the genus level, were significantly overrepresented in one or more soil management conditions. The OTU (NCROTU441) assigned to a *Claroideoglomus* unidentified species was significantly overrepresented in the tilled plots in the soil with crop rotation. This OTU also resulted as indicator species of the site group combination T (R vs. NR).

The OTU104, assigned to the family Glomeraceae, was significantly overrepresented in tilled soil both with and without crop rotation. Three other unidentified OTUs, all assigned to the Glomeraceae family, were overrepresented in the no-tilled soil without crop rotation (OTU14, NCROTU740, NCROTU674). The Glomeromycota that includes only one class, Glomeromycetes, comprehend fungi forming arbuscular mycorrhizas symbioses (AMF) with the roots of the majority of plant species (>80%) (Hempel et al., 2007; Giovannetti, 2008). AMF abundance and diversity can be sensibly reduced by intensive land use associated with mineral fertilizer and tillage (Pellegrino et al., 2020). Moreover, the identity of crops shaped the composition and structure of the communities of AMF in agricultural soils. Agricultural soil management can homogenize AFM communities by favoring ruderal and disturbance-tolerant taxa and the destruction of spatial heterogeneity. Glomeromycota in the soil under study, independent of the kind of management, was 1% of total fungal abundance. It is a figure that, compared to other soils, is not surprising. Misra et al. (2019) found 0.2–2% of Glomeromycota in the bulk soil and higher levels in rhizosphere soil compared to bulk soil of two aromatic plants cultivated in continuous cropped fields. Also, in the rhizosphere soil of a long-term continuous cropping system, Glomeromycota accounted for 1.21% of the dominant phylum, according to the findings of Zhu et al. (2018). Sugiyama et al. (2010) found a low abundance of Oomycota and Glomeromycota (0.4% of Oomycota sequences and 0.1% of Glomeromycota sequences) in soil from potato fields, and Jumpponen et al. (2010) found low levels of Glomeromycota (1%) in a tallgrass prairie soil. However, some studies on agricultural soils showed very different values, as in Xu et al. (2017). They investigated AMF communities in Chinese agricultural soils using high-throughput sequencing technologies and found that Glomeromycota accounted for 60.1% of the total fungal sequences in the rhizosphere soil.

## Conclusion

Tillage practices shape soil properties and impact soil fungal communities. Higher biodiversity of saprotrophic fungi characterized soil sites with low disturbance. The negative correlation between the biodiversity of ectomycorrhizal and saprotrophic fungi underlined how their mutual exclusion could be due to a substrate-mediated niche partitioning or by space partitioning.

Where the soil was subject to tillage, fungal species appeared evenly distributed. In contrast, there was higher spatial variability in the absence of tilling, with fungal taxa distributed according to a small-scale pattern, corresponding to micro-niches that probably remained undisturbed and heterogeneously distributed.

Many differentially represented OTUs in all the conditions investigated were unidentified species or OTUs matching at high taxa level (i.e., phylum, class, order). This outcome stresses how little is known about fungi in agricultural soils and how many fungal species, probably fundamental to the homeostasis of agricultural productivity, are yet unknown or have not yet been sequenced and simultaneously associated with species known to science.

Among the fungi with key roles in all the investigated conditions, there were several yeast species known to have pronounced endemism in soil. Some fungal species emerged as either indicator of a kind of management or as strongly associated with a specific condition. In particular, *Minimedusa polyspora*, which was overrepresented in the tilled soil, independent of crop rotation, and the yeast *Saitozyma podzolica*, one of the top indicator species of the non-rotated plots.

No-tillage in the long term promoted the biodiversity of the fungal community through greater fragmentation of the environment into micro-niches that were stable enough to support species with different functionalities. Understanding how agricultural practices influence fungal functional diversity and distribution in the soil can guide agricultural systems that enhance positive soil functions, including soil C sequestration, pathogens control and nutrients cycling.

## Data Availability Statement

The datasets presented in this study can be found in the Supplementary Materials and the sequences are deposited in the European Nucleotide Archive (ENA, https://www.ebi.ac.uk/ena) with the accession number PRJEB41172.

## Author Contributions

LC and LO contributed equally to the study, conducted the molecular analyses, processed and interpreted the sequencing data, and wrote the manuscript. AT performed the statistical analyses, interpreted the soil chemical data, and wrote the manuscript. MM, BP, and AM conducted the sampling, participated in soil analyses, and processed the soil chemical data. RF conceived the design of the study and supervised the field trials and the project. FP conceived the design of the study, supervised the project, interpreted sequencing data, and wrote the manuscript. All authors contributed to the article and approved the submitted version.

## Conflict of Interest

The authors declare that the research was conducted in the absence of any commercial or financial relationships that could be construed as a potential conflict of interest.

## Footnotes

1 https://github.com/UMNFuN/FUNGuild
