# Supplementary material for: How Tillage and Crop Rotation Change the Distribution Pattern of Fungi
Source: Front Microbiol. 2021 Jun 17;12:634325. doi: 10.3389/fmicb.2021.634325 (PMC8247931; doi:10.3389/fmicb.2021.634325)

Supplementary Material

1 Description of Online Resources

Supplementary Table 1 (OTUtable): Raw reads were filtered using Trimmomatic (Bolger, 2014) to remove the adapters and low-quality reads using a quality cutoff of 20 in 24bp sliding windows. Paired-end reads were merged using Pear (Zhang et al, 2014). Reads were clustered into OTUs based on a 97% sequence similarity using the "open reference OTU picking protocol" implemented in the QIIME toolkit (Caporaso et al., 2010). Low abundant OTUs were removed, filtering out all the OTUs showing less than three sequences. Samples were normalised to the size of the smallest sample (67,511 sequences each) to allow comparison. Taxonomy was assigned using the UNITE database. After normalisation, a total of 1646 OTUs were obtained. QIIME1 have assigned the taxonomic data to each OTU blasting the representative sequences against the UNITE database using by default the minimum per cent identity of 90%. The blast E-values for each OTU, filtered for significance, are shown in a dedicated sheet of this excel file.

Supplementary Table 2 (OTUs distribution): Taxonomic distribution of fungal OTUs. The OTUs were assigned to 7 Phyla and 284 different genera.

Supplementary Table 3 (Shannon guilds): FUNGuild (https://github.com/UMNFuN/FUNGuild) database was used to assign ecological guilds to the OTUs (Nguyen et al., 2016). Shannon (H) and Simpson (1-D) indexes were calculated using the Vegan R package. The normal distribution of data within each condition was verified using the Shapiro Wilk test in R (V3.4.4). The ANOVA test and Tukey's post hoc test were performed using the R package *Stat* 3.6.2. The Kruskal Wallis and the post hoc Dunn test with Benjamini Hochberg correction were performed using the R package *dunn.test v* 1.3.5.

Supplementary Table 4 (Alpha Diversity Saprophytes): Result of Tukey's post hoc test for the alpha diversity of saprophytic fungi. FUNGuild (https://github.com/UMNFuN/FUNGuild) database was used to assign ecological guilds to the OTUs (Nguyen et al., 2016). FUNGuild database divides the OTUs into 16 categories. However, for the alpha diversity analysis, we grouped within the broad category of saprotrophic fungi all the OTUs annotated respectively as leaf saprotroph, undefined saprotrophs, dung saprotroph and wood saprotrophs. Within the "saprotroph" class, we also considered the Mortierellaceae that were annotated by FUNGuild both as endophyte and saprotroph.

Supplementary Table 5 (EdgeR): The spreadsheet reports the logFC, the P-value, and the annotation of the OTUs found significantly differentially abundant with EdgeR. Different abundant OTUs in pairwise comparison between treatments were identified using the edgeR package V 3.28.1 (Robinson et al., 2010). The statistical significance threshold was defined by an adjusted P-value ≤ 0.05 and an absolute log2 fold change > 2.

Supplementary Table 6 (IndVal indicator species): The specificity of an OTU to a given treatment was determined using the Dufrene-Legendre indicator value (IndVal) index (Dufrêne and Legendre, 1997) using the *labdsv* package in R. "Indicator value" indices assess the predictive value of a species as an indicator of a combination of site groups. The indicator value index was calculated as the product of two quantities, A and B. "A" is the probability of a site being a member of a given site-group combination (T/NT soil combined with R/NR crops) when the species has been found at that site. Quantity "B" informs how frequently the species is found at sites of the site-group combination under study. The "Correlation indices" assess the positive or negative preference of the species for the

environmental conditions prevailing within sites belonging to the site-group combination, compared to the remaining sites.

2 Supplementary Figure S1

These rarefaction curves show how the number of OTUs found in each soil fungal communities increases with increasing the sequencing coverage.

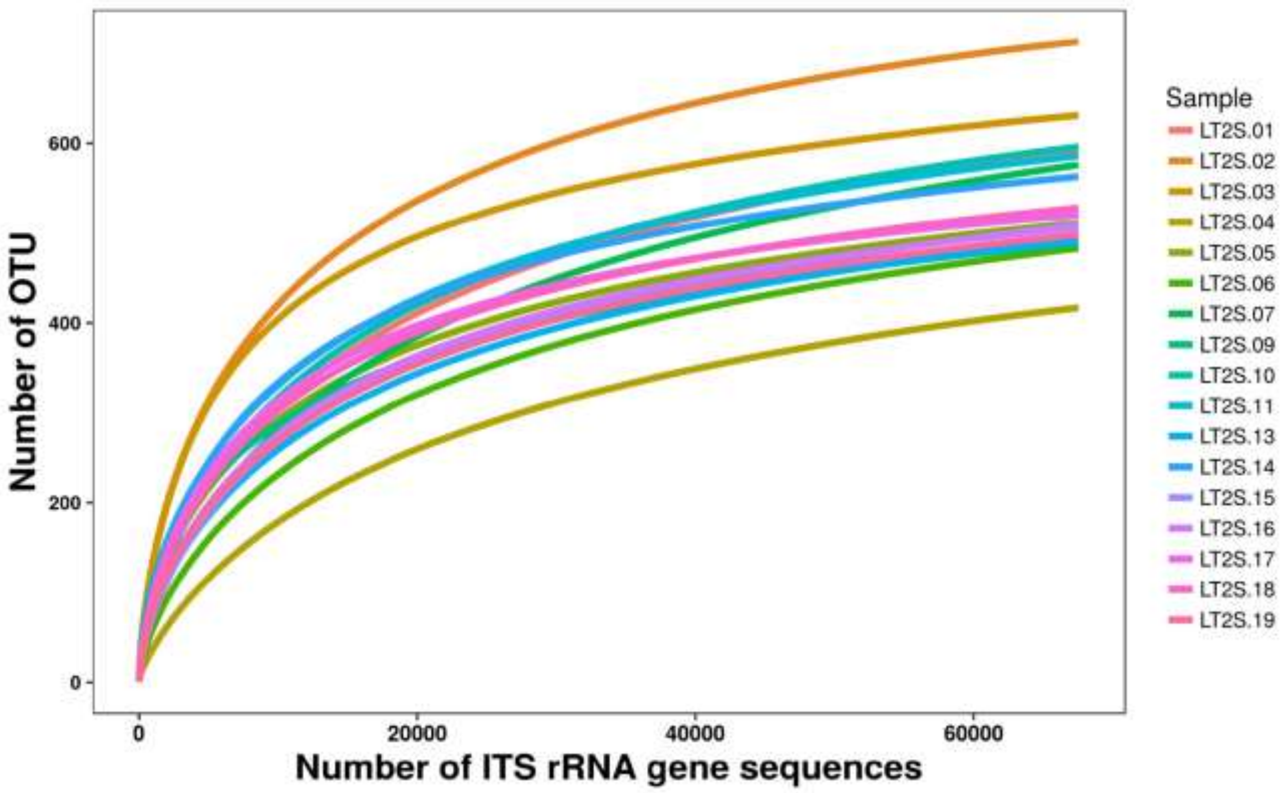

3 Supplementary Figure S2

This scatterplot shows Pearson's correlation between the abundance (number of sequences) of fungal species of the genus *Mortiriella* with the bioavailable soil phosphorus (mg/kg).

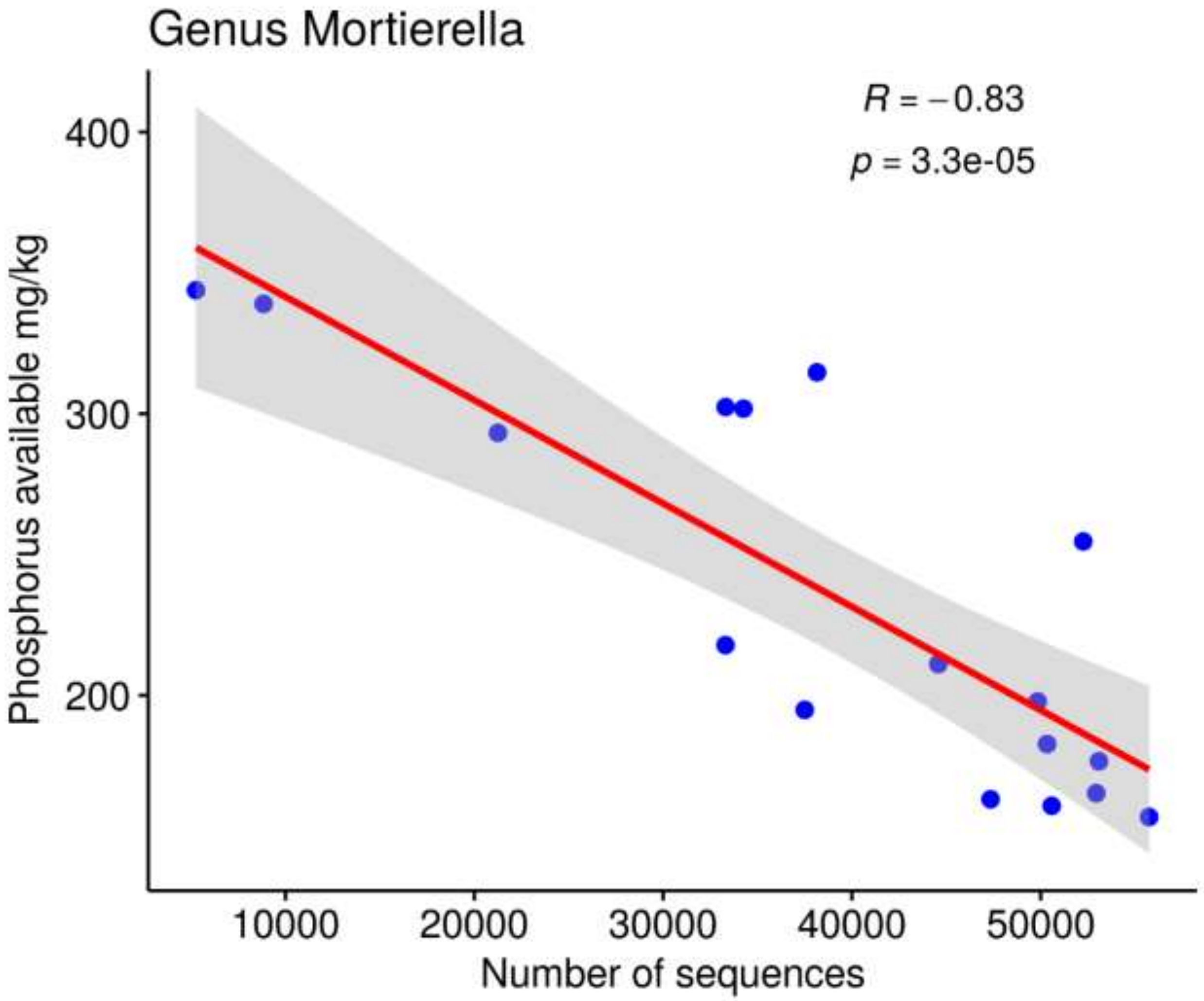

Supplement: Supplementary file 1 [file Data_Sheet_1.pdf]
